# The inventory of camel feed resource and the evaluation of its chemical composition in south‐east rangelands of Ethiopia

**DOI:** 10.1002/vms3.471

**Published:** 2021-03-22

**Authors:** Matiwos Habte, Mitiku Eshetu, Dereje Andualem, Melesse Maryo, Abiyot Legesse

**Affiliations:** ^1^ Africa Center of Excellence for Climate Smart Agriculture and Biodiversity Conservation Haramaya University Dire Dawa Ethiopia; ^2^ Department of Animal and Range Sciences Dilla University Dilla Ethiopia; ^3^ School of Animal and Range Sciences Haramaya University Dire Dawa Ethiopia; ^4^ Ethiopian Biodiversity Institute Addis Ababa Ethiopia; ^5^ Department of Geography and Environmental Studies Dilla University Dilla Ethiopia

**Keywords:** browse species, digestibility, feed quality, forage, livestock

## Abstract

**Background:**

Evaluations of available camel feed nutritive value are relevant to generate evidence on further camel feed improvements and find out the components to be supplemented.

**Objective:**

This study aim to evaluate seasonal variations on chemical composition of selected camel feed in semi‐arid regions of south‐east Ethiopia.

**Methods:**

Samples of edible portions from 15 browse species were collected during the dry and wet seasons, and their chemical compositions were analysed.

**Results:**

The crude protein (CP), in vitro dry matter digestibility (IVDMD) and relative feed value (RFV) of evaluated browse species were higher (*p* < .01) in wet season than the dry season except for *Acacia asak*, *Ipomoea donaldsonii* and *Acacia mellifera*. Nonetheless, the neutral detergent fibre (NDF) and acid detergent fibre (ADF) contents were higher (*p* < .01) in the dry season except for *A*. *asak* and *I. donaldsonii*. Moreover, *A*. *asak*, *I*. *donaldsonii* and *A*. *mellifera* were the browse species with higher (*p* < .01) RFV, IVDMD and CP but lower NDF and ADF during the dry season than the wet season, and these species are qualified as good‐quality forage. Thus. *Barleria spinisepala* were better to use in both seasons, but browse species like *I*. *donaldsonii* and *A*. *asak* in dry season were ranked as best quality roughage.

**Conclusion:**

At richest level on vital components (CP and fibres), these species can serve as well ruminant diets, like for camel. Further investigations based on animal trials are needed in order to confirm the classification standards of feed quality used in this study.

## Graphical abstract

Browse species which potentially utilized by camel was assessed Seasonal factors which influence camel feed chemical composition was described some of the excellent quality forage were proposed for inclusion in animal feed.

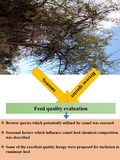

## 1 INTRODUCTION

Animal feed resources are mainly derived from the natural pasture in Ethiopia (CSA, 2012). Agroecology and land use/cover types determine a potential contribution to palatable livestock feed resources (Bediye et al., 2001; Madsen et al., 2008). This potential contribution showed a declining trend in the last few decades, due to the expansion of cropland cover and invasion of unpalatable woody vegetation into the grasslands (Gebremedhin et al., 2009). Thus, potential contributions of grassland cover to livestock feed resources have been gradually decreasing and became less indispensable in arid and semi‐arid regions (Thornton, 2010). According to Konuspayeva (2007), the livestock feeds derived from bush/shrubland vegetation cover are gradually increasing in arid and semi‐arid regions of Ethiopia.

Alemayehu et al., (2017) indicated that livestock feed quality is the major bottleneck for livestock production in semi‐arid regions of Ethiopia. Following the results of McDonald et al., (1995), the quality of forage has been determined by its chemical and biological nutrients, which directly influence the digestibility and feed intake; consequently, milk and meat productivity were affected by consuming low crude protein (CP) and high contents of fibre. The seasonal variation of browse species, nutritional composition and forage quality is the primary concern (Chalchissa et al., 2014). As an example, the CP content of browse species was higher in the wet season and dropped down in the dry season (Melaku et al., 2010; Yayneshet et al., 2009). Quality and availability of browse species vary with agroecology, rainfall and temperature patterns, which limit biomass production and nutritive value (Melaku et al., 2010). Main browses species of natural pasture are useful for animal feeding in changing eco‐environments of semi‐arid regions. The primary contributors of browse feed resources such as bush, shrub and woody vegetation remain evergreen throughout the year with better forage quality when grasses dry out (Aregawi et al., 2008).

Temperature and rainfall affect forage quality through ecophysiological changes of plant species or direct influence on feed digestibility (Ball et al., 2001). Plants successively undergo ecophysiological changes in response to heat stress, low precipitation and soil water scarcity. Accordingly, climatic extremes lead to slow rate of plant maturation and decrease plants' water content and the leaf‐to‐stem ratio (high lignin and cell wall contents) which strongly affects feed digestibility (Collins, 1988; Rivera & Parish, 2010; Stone et al., 1960). High temperature increases plants' lignification process and decreases ruminants' voluntary feed intake as response to thermoregulation mechanism.

Studies related to the environmental effects on forage quality and availability in tropics in general and in Ethiopia in particular have been conducted mainly on few legume species such as *Desmodium ovalifolium* (herbaceous legume) and *Calliandra calothyrsus* (shrub legume) (Chou et al., 2008; Dumont et al., ,^2014^, ^2015^; Hidosa & Guyo, 2017). Furthermore, Madalcho et al., (2019) identified 50 species of trees and shrub plants that have potentially been used as camel feed resources in east and south‐east rangelands of Ethiopia. Consequently, there are minimal information produced about quality parameters and nutritional composition of browse feed resources in arid and semi‐arid condition. Melaku et al., (2010) have evaluated the quality parameters of few browse species that have potentially be utilized by dromedaries. However, dromedaries tend to browse a wide variety of plant species in open rangeland condition (Mirkena et al., 2018).

Moreover, Moges et al., (2016) attempted to address the gap on low‐quality camel feed resources in the rangeland through feed supplementation on top of free‐ranging. The finding focused on feed supplementation of concentrates and urea‐treated roughage on top of free‐ranging because the available forage species have been depleting in quality. However, the findings lack the assessment and evaluation of seasonal nutritive variations of feed resources that have been potentially utilized by browser livestock animals in semi‐arid region. Therefore, assessments of available camel feed resources and evaluation of its nutritive value are relevant to generate evidence on further camel feed improvements and find out the components to be supplemented to cope up with the impacts of declining feed quality. This study aim to evaluate seasonal variations on chemical composition of selected camel feed in semi‐arid regions of south‐east Ethiopia.

Seasons and browse species can influence chemical composition and forage quality of camel feed resources.

## 2 MATERIAL AND METHODS

### 2.1 Description of the study area

The study was conducted in the semi‐arid area of East Guji Zone, south‐east Ethiopia. It is located between 4° 38′ 55″ N and 5° 33′ 7″ N latitude and 39° 9′ 25″ E and 39° 58′ 37″ E longitude and cover about 742,644 ha. The locations are categorized as a pastoral and agro‐pastoral region that belongs to the semi‐arid lowland agroecological zone. The altitude of the study districts ranges between 1,370 and 1,650 m above sea level (m.a.s.l). The annual temperature of the area varied from 24 to 30°C with a mean annual rainfall of 526.75 mm. The pattern of the rain is bimodal with the primary wet season (Ghana) contributing about 60% of yearly rainfall which extends from March to May, while dry season ranges from December to February (Abate, 2016).

### 2.2 Inventory of camel feed resources

#### 2.2.1 Sampling procedures and design

Three study districts were randomly selected from the five pastoral and agro‐pastoral districts of East Guji Zone based on the drawing lots procedure indicated in Gomez and Gomez (1984). The study considered two traditionally classified geographical locations, namely, *Golba* (covers the altitude below 1,450 m.a.s.l) and *Dida* (the altitude up to 1,650 m.a.s.l). Three *kebeles* (the smallest administrative unit of Ethiopian Government) were randomly selected from each location. Accordingly, Hadhessa, Qoratti and Siminto *kebeles* were selected from *Dida* location, and Kalada, Gofi Ambo and Nura Umba were selected from *Golba* study location.

#### 2.2.2 Focus group discussion

Focus group discussion (FGD) was conducted to obtain a general overview of the camel feed resources and identify potential browse species that camel can have access in both dry and wet seasons. Thirty‐six participants from both sexes representing all groups of the community, locations, education level and the household heads with above 35 years were selected to identify available camel feed resources in the study area following Geilfus (2008) procedure, because camel raisers with more than 35 years old perceived as better in camel feeding experience. Six FGDs, one in each *kebeles* consisting of eight participants, were employed. The FGD were selected based on their experiences on camel raising, feeding and moving with a camel in the rangeland. Furthermore, the discussion was conducted with the local language (Afan Oromo) as the study locations are solely Oromo's ethnic group.

#### 2.2.3 Description of the selected browse species

The choice of collected browse species (Table 1) depends on its availabilities in the area, contributions in camel feeding and preference by camels as indicated by FGDs.

**TABLE 1:** Description of the analysed forage species scientific, local and their family names

S. no	Family	Scientific name	Local name
1	Acanthaceae	*Barleria spinisepala*	Qilxiphee
2	Anacardiaceae	*Lannea rivae*	Andaraka
		*Rhus ruspolii*	Daboobeessaa
3	Balantiaceae	*Balanites rotundifolia*	Baddana
4	Burseraceae	*Commiphora erythraea*	Agarsuu
		*Commiphora africana*	Qaayyoo
5	Convolvulaceae	*Ipomoea donaldsonii*	Dhaallaa
6	Cyclocheilaceae	*Asepalum eriantherum*	Gurbii Aadii
7	Ebenaceae	*Euclea divinorum*	Miessaa
8	Fabaceace	*Acacia mellifera*	Saphaansa Gurraacha
		*Acacia bussei*	Halloo
		*Acacia asak*	Bokossaa
		*Dalbergia microphylla*	Walchamala
		*Tephrosia pentaphylla*	Birreessa
9	Tiliaceae	*Grewia tembensis*	Dheekkaa
		*Grewia evolute*	Arooressa
Total	9	16	16

#### 2.2.4 Sample collection and preparation

The browse species samples were collected in the wet (March to May) and dry (December to February) seasons. This study is based on the identified flora of southern Ethiopian rangeland by Gemedo‐Dalle et al., (2005). All edible portions of collected browse species samples were labelled and dried for the analysis of chemical composition. Samples of the same feed type were bulked together on a seasonal basis and then thoroughly mixed and subsampled following the method indicated by Herrman (2001) and Feeding‐Stuffs (1988). The edible, healthy portions were sampled from 12 representative plants of the selected browse species, weighed immediately after collection with digital sensitive balance and oven‐dried at 65 C for 72 hr. The dried samples were ground pass 1 mm Wiley sieve size and used for determination of chemical composition and in vitro dry matter digestibility (IVDMD). The sieved samples were kept in airtight containers pending analysis for chemical composition.

### 2.3 Determination of nutritive value of browse species

Feed samples were analysed for dry matter (DM), ash and CP according to the standard procedures for feedstuffs analysis (AOAC, 1990). Neutral detergent fibre (NDF) and acid detergent fibre (ADF) and acid detergent were analysed by the method of Van Soest et al., (1991). The method of Tilley and Terry (1963) as modified by Van Soest and Robertson (1985) was used to determine IVDMD. The donor animals of the rumen liquors used for IVDMD analysis were five finished bucks slaughtered at Dilla municipality abattoir. The rumen digesta was collected from the reticulum in an insulated thermos flask, sealed and transported immediately to the laboratory. The rumen liquor was filtered through two layers of gauze cloth, mixed with each species on a volume basis, flushing with CO_2_, and stored in a pre‐warmed thermos for approximately 20 min (until use). Total digestible nutrient (TDN) value of the selected browse species was determined using the formula suggested by Reid et al., (1952), with 60% digestion coefficient. The digestible dry matter (DDM), dry matter intake (DMI) and relative feed value (RFV) were determined using the index recommended by Rivera and Parish (2010), Jeranyama and Garcia (2004) and Kiraz (2011).

*F* = *M* (0.01 + (0.000125 × *E*)), where *F* is conversion factor, *M* is the percent of organic matter (OM) on DM basis and *E* is the ether extract (EE) as per cent of the OM.

$$\text{Total digestible nutrient}\;\left(\text{TDN}\%\right)=F\times 60\%.$$

$$\text{Digestible dry matter}\;\left(\text{DDM}\%\right)=88.9‐\left(0.779\times \text{ADF}\right)\;\left(\text{basis}\;\%\;\text{DM}\right).$$

$$\text{Dry matter intake}\;\left(\text{DMI}\right)=120/\text{NDF}\;\left(\text{basis}\;\%\;\text{DM}\right).$$

$\text{Relative feed value}\;\left(\text{RFV}\right)=\text{DDM}\times \text{DMI}/1.29$. The RFV was compared with full bloom alfalfa (reference feed), which is assigned an RFV of 100 (Rivera & Parish, 2010; Undersander et al., 2002).

### 2.4 Statistical analysis

The analysis of variance (ANOVA) was conducted using the general linear model (GLM) procedure of SAS (2010) for Windows. ANOVA model statement used to investigate the effects of season on feed chemical composition of browse species and difference of feed quality. The model used to estimate the variance component was two‐way ANOVA procedure, which considered season, species and interaction effects as different factors.

$$\text{Model}:Y_{\mathrm{ijk}}=\mu +{\text{FS}}_{i}+S_{j}+{\left(S*\text{FS}\right)}_{\mathrm{ij}}+e_{\mathrm{ijk}}.$$

where *Y_ijk_* is measurements of feed chemical composition in *i*th feed species at *j*th season; *μ* is the fixed effects of feed chemical composition in *i*th feed species at *j*th season; *FS_i_* is effects of feed species; *S_j_* is fixed effects of season; (S*FS)_ij_ is interaction effects of season and feed species; and *e_ijk_* is residual.

Mean separation was employed using Duncan multiple range tests (Duncan, 1955). Moreover, Pearson product‐moment correlation was used to measure the association between weather condition and camel physiological response (Chee, 2013). All results were presented as means ± standard error of means (means ± SE).

## 3 RESULT AND DISCUSSION

### 3.1 Inventory of camel feed resources in south‐eastern Ethiopia

Focus group discussion have identified about 49 browse plant species that can potentially be utilized by camel during the dry and wet seasons in eco‐environments of the study area. The identified species of feed resources were grouped into 24 families (Table 2).

**TABLE 2:** Identified camel feed resources in south‐eastern Ethiopian rangeland

No	Family name	Botanical name	Local name	Edible plant part
1	Acanthaceae	*Blepharispermum pubescens* S. Moore	Beenyaa	Leaf
		*Barleria spinisepala* E. A. Bruce	Qilxiphee	Stem and leaf
	Anacardaceae	*Rhus ruspolii* Engl.	Dabobessa	Leaf
2		*Rhus ruspolii* Engl.	Daboobeessaa	Leaf
		*Lannea rivae* (Chiov.) Sacleux	Andaraka	Leaf
3	Apiaceae	*Steganotaenia araliaeae* Hochst.	Luqaaluqqee	Leaf
4	Asparagaceace	*Asparagus falcatus* L.	Sareetii	Whole
5	Asteraceae	*Aspilia mossambicensis* (Oliv.) H. Willd.	Adaa	Leaf
6	Balanitaceae	*Kleinia squarrosa* Cufod.	Xixiixxuu	Leaf
		*Balanites rotundifolia* (Van Tiegh.) Blatter	Baddana	Leaf
7	Burseraceae	*Commiphora erythraea* (Ehrenb.) Engl.	Agarsuu	Leaf and seed
		*Boswellia microphylla* Chiov.	Ilka buqqisaa	Leaf
		*Boswellia neglecta* S. Moore	Dakkara	Leaf
		*Commiphora africana* (A. Rich.) Engl	Ammessa Adii	Leaf
		*Commiphora schimperi* (Berg) Engl.	Hammeessa qayyoo	Leaf
		*Commiphora kua* (R. Br. ex Royle) Vollesen	Callaanqaa	Leaf
8	Commelinaceae	*Commelina africana* L.	Qaayyoo	Whole
9	Convolvulacaee	*Ipomoea donaldsonii* Rendle	Dhaallaa	Leaf
10	Cyclocheilaceae	*Asepalum eriantherum* (Vatke) Marais	Gurbii Aadii	Stem and leaf
11	Dracaenaceae	*Sansevieria ehrenbergii* Schweinf. ex Baker	Cakkee	Whole
12	Ebenaceae	*Euclea divinorum* Hiern	Miessaa	Leaf
	Euphorbiaceae	*Acalypha fruticosa* Forssk.	Dhirrii booranoo	Leaf
13		*Phyllanthus sepialis* Mu¨ll. Arg	Dhirrii warseessoo	Leaf
14	Fabaceace	*Acacia goetzei* Harms	Burraa	Leaf
		*Dichrostachys cinerea* Wight et Arn	Jirimee	Leaf
		*Acacia etabaica* Schweinf	Alqabeessa	Leaf
		*Acacia asak* (Gemedo Dalle No. 289)	Bokossaa	Leaf
		*Tephrosia vogelii* Hook. f.	Birreessa	Leaf
		*Acacia hockii* De Willd.	Dabaso	Leaf
		*Acacia brevispica* Harms	Hamarresssa	Leaf
		*Acacia mellifera* (Vahl.) Benth	Saphaansa Gurraacha	Leaf
		*Acacia bussei* Harms ex Sjostedt	Halloo	Leaf
		*Acacia nilotica* Willd. ex Del.	Burquqqee	Leaf
		*Acacia drepanolobium* Harms ex Sjoestedt	Fulleessa	Leaf
		*Dalbergia microphylla* Chiov.	Walchamala	Leaf
		*Acacia senegal* Willd.	Saphansa Diimaa	Leaves and succulent branches
		*Acacia seyal* Del.	Waaccuu	Leaf
		*Acacia tortilis* (Forssk.) Hayne	Dhaddacha	Leaf
15	Ochnaceae	*Ochna inermis* (Forssk.) Schweinf	Aqalqabaa	Leaf
16	Sapindaceae	*Dodonea angustifolia* L. f.	Dhitacha	Leaf
17	Simaroubaceae	*Kirkia burgeri* Stannard. ssp. Burgeri	Bisdhugaa	Leaf
18	Solanaceace	*Solanum giganteum* Jacq.	Iddii loon	Leaf
19	Sterculiaceae	*Harmsia sidoides* K. Schum	Qaxxee	Leaf
20	Tiliaceae	*Grewia evolute* Juss.	Arooressa	Leaf
		*Grewia villosa* Willd.	Ogomdii	Leaf
		*Grewia tembensis* Fresen.	Dheekkaa	Leaf
		*Grewia penicillata* Chiov.	Ogomdii dhiirsoba	Leaf
21	Verbenaceae	*Premna schimperi* Engl.	Xaaxessaa	Leaf and stem
22	Vitaceae	*Cissus aphyllantha* Gilg.	Cophii soodduu	Whole

Trees and shrubs such as *Acacia asak*, *Acacia lahai*, *Acacia oerfota*, *Acacia tortilis*, *Albizia amara*, *Dobera glabra*, *Ficus glumosa*, *Ziziphus spina‐christi*, *Terminalia brownii*, *Ximenia americana* and *Rhus natalensis* plants are the primary feed resources for camel in the Horn of Africa (Aregawi et al., 2008; Lu et al., 2012; Yagil, 1982). Cattle and sheep species do not readily utilize most of the preferred plant species by camel because they are bitter and thorny (Lu et al., 2012).

### 3.2 Chemical composition and nutritional quality of camel feed resources

#### 3.2.1 Dry matter

The DM contents of analysed browse forage species were varied from 85.6% in *Lannea rivae* to 93.3% in *Balanites rotundifolia* during the dry season and 86.7% in *L*. *rivae* to 94.6% in *A*. *asak* during the wet season. The DM contents of all considered browse species showed statistically insignificant (*p* > .05) variation across season except for *L*. *rivae* and *A*. *asak* (Table 3). Moreover, FGDs revealed the substantial contributions of *Grewia tembensis*, *L. rivae*, *Commiphora erythraea*, *Dalbergia microphylla and Euclea divinorum* species in camel feeding. The range of DM content observed in this study at both seasons corresponds with the finding of Kuria et al., (2012) who reported 91.1% of mean DM contents of browse species mainly preferred by camel. Similarly, Dalle (2020) and Nsubuga et al., (2019) revealed that the DM concentrations of edible browse species range from 88% to 93% in arid and semi‐arid regions, which is consistent with the current finding. Moreover, Melaku et al., (2010) have reported 90.6% for mean DM content of plant species available in semi‐arid regions of northern Ethiopia, indicating that DM constituents of browse forage species did not vary with location.

**TABLE 3:** Chemical composition and nutritional quality of selected browse species in south‐eastern rangeland of Ethiopia (mean ± SE)

Season	Species	CP (%DM)	Ash (%DM)	OM (%DM)	RFV
Dry	*Acacia bussei*	7.02 ± 0.1^m^	9.97 ± 0.1^cdef^	90.03 ± 0.1^f‐i^	88.02 ± 1.0^p^
	*Acacia mellifera*	21.96 ± 0.4^b^	1.34 ± 0.1^i^	98.66 ± 0.1^a^	174.25 ± 3.6^b^
	*Acacia asak*	18.28 ± 0.2^ef^	8.11 ± 0.1^defgh^	91.89 ± 0.1^c‐‐h^	154.78 ± 2.6^ef^
	*Asepalum eriantherum*	9.09 ± 0.1^kl^	7.17 ± 0.0^defgh^	92.83 ± 0.0^b‐e^	104.86 ± 1.5^mno^
	*Balanites rotundifolia*	9.60 ± 0.3^kl^	7.38 ± 0.1^defgh^	92.62 ± 0.1^b‐g^	108.81 ± 4.6^lmn^
	*Barleria spinisepala*	20.59 ± 0.3^be^	9.44 ± 0.2^cdefg^	90.56 ± 0.2^d‐j^	169.42 ± 3.3^bc^
	*Commiphora erythraea*	10.24 ± 0.2^kl^	4.69 ± 0.0^hi^	95.31 ± 0.0^b^	112.26 ± 2.6^klm^
	*Dalbergia microphylla*	12.25 ± 0.1^j^	5.62 ± 0. 0^fghi^	94.38 ± 0.0^bc^	117.08 ± 1.7^ik^
	*Euclea divinorum*	7.00 ± 0.1^m^	6.78 ± 0.1^efgh^	93.22 ± 0.1^bcd^	97.17 ± 2.3^o^
	*Grewia evolute*	10.49 ± 0.1^k^	9.36 ± 0.1^cdefg^	90.64 ± 0.1^d‐j^	111.31 ± 1.1^lmn^
	*Grewia tembensis*	17.07 ± 0.2^fg^	9.76 ± 0.0^cdef^	90.24 ± 0.0^e‐j^	147.20 ± 2.1^fg^
	*Ipomoea donaldsonii*	19.28 ± 0.3^cde^	10.01 ± 0.1^cdef^	89.99 ± 0.1^g‐j^	158.44 ± 3.5^de^
	*Lannea rivae*	12.32 ± 0.2^ij^	8.02 ± 0.1^defgh^	91.98 ± 0.1^c‐h^	113.91 ± 3.2^klm^
	*Rhus ruspolii*	12.46 ± 0.1^ij^	10.12 ± 0.0^cde^	89.88 ± 0.0^h‐j^	120.96 ± 1.5^jk^
	*Tephrosia vogelii*	10.47 ± 0.1^k^	7.32 ± 0.1^defgh^	92.68 ± 0.1^b‐j^	110.62 ± 2.1^lmn^
	Mean	13.21 ± 0.7^b^	7.67 ± 0.4^b^	92.33 ± 0.4	125.94 ± 4.0^b^
Wet	*Acacia bussei*	20.34 ± 0.1^c^	5.58 ± 0.2^fghi^	94.42 ± 0.2^bc^	165.82 ± 0.9^bcd^
	*Acacia mellifera*	21.92 ± 0.0^b^	10.13 ± 0.2^cde^	89.88 ± 0.2^hij^	170.82 ± 0.5^bc^
	*Acacia asak*	9.64 ± 0.2^kl^	6.72 ± 0.1^efgh^	93.28 ± 0.1^bcd^	109.24 ± 2.5^lmn^
	*Asepalum eriantherum*	16.92 ± 0.1^fg^	10.78 ± 0.1^defgh^	89.22 ± 0.1^ij^	144.61 ± 1.1^gh^
	*Balanites rotundifolia*	14.76 ± 0.3^h^	7.93 ± 0.1^a^	92.07 ± 0.1^c‐h^	132.80 ± 3.0^i^
	*Barleria spinisepala*	23.56 ± 0.7^a^	20.68 ± 0.6^defgh^	79.32 ± 0.6^m^	208.46 ± 7.5^a^
	*Commiphora erythraea*	18.88 ± 0.0^de^	7.78 ± 0.2^defgh^	92.22 ± 0.2^c‐h^	158.20 ± 0.5^de^
	*Dalbergia microphylla*	14.21 ± 0.1^h^	7.26 ± 0.0^ghi^	92.74 ± 0.0^b‐f^	128.26 ± 1.6^ij^
	*Euclea divinorum*	8.98 ± 0.1^l^	4.98 ± 0.2^ghi^	95.02± 0.2^b^	101.82 ± 1.0^o^
	*Grewia evolute*	24.27 ± 0.3^a^	11.39 ± 0.0^bcd^	88.62 ± 0.0^jk^	210.65 ± 3.4^a^
	*Grewia tembensis*	20.10 ± 0.4 cd	10.26 ± 1.7^bcde^	89.74 ± 1.7^hij^	163.04 ± 3.9^cde^
	*Ipomoea donaldsonii*	16.46 ± 0.3^g^	14.63 ± 1.2^b^	85.37 ± 1.2^l^	136.21 ± 3.2^hi^
	*Lannea rivae*	13.42 ± 0.1^hij^	13.26 ± 3.6^bc^	86.74 ± 3.6^kl^	127.17 ± 1.7^ij^
	*Rhus ruspolii*	14.67 ± 0.4^h^	4.83 ± 1.2^hi^	95.17 ± 1.2^b^	134.23 ± 4.8^i^
	*Tephrosia vogelii*	13.74 ± 0.4^hi^	5.58 ± 0.3^fghi^	94.42 ± 0.3^bc^	128.30 ± 4.9^ji^
	Mean	16.79 ± 0.7^a^	9.45 ± 0.7^a^	90.55 ± 0.7	147.98 ± 4.7^a^
*p‐*Value	Feed species	<0.0001	<0.0001	<0.0001	<0.0001
	Season	<0.0001	<0.0001	<0.0001	<0.0001
	Interaction	<0.0001	<0.0001	<0.0001	<0.0001

Season	Species	DM	EE (%DM)	TDN	IVDMD
Dry	*Acacia bussei*	92.76 ± 0.7^ab^	12.28 ± 1.2^a‐e^	62.31 ± 0.8^d‐h^	39.52 ± 0.3^n^
	*Acacia mellifera*	91.38 ± 1.5^ab^	13.55 ± 0.7^abc^	69.23 ± 0.5^a^	60.53 ± 1.0^abc^
	*Acacia asak*	90.42 ± 1.2^ab^	11.69 ± 0.5^a‐e^	63.19 ± 0.4^c‐g^	54.72 ± 0.7^def^
	*Asepalum eriantherum*	91.24 ± 0.9^ab^	3.87 ± 1.0^ij^	58.40 ± 0.7^ijk^	42.82 ± 0.4^lmn^
	*Balanites rotundifolia*	93.29 ± 2.8^ab^	2.71 ± 0.7^j^	57.45 ± 0.4^jk^	43.42 ± 1.3^lmn^
	*Barleria spinisepala*	89.56 ± 1.4^ab^	5.37 ± 1.0^g‐j^	57.98 ± 0.6^ijk^	60.12 ± 0.9^bc^
	*Commiphora erythraea*	88.52 ± 1.5^ab^	11.31 ± 1.4^a‐f^	65.27 ± 1.0^bcd^	44.00 ± 0.8^lmn^
	*Dalbergia microphylla*	91.29 ± 1.0^ab^	13.51 ± 1.0^abc^	66.19 ± 0.7^bc^	46.21 ± 0.5^jkl^
	*Euclea divinorum*	88.59 ± 1.4^ab^	8.67 ± 0.5^b‐i^	61.99 ± 0.4^rfg^	40.72 ± 0.7^mn^
	*Grewia evolute*	87.10 ± 0.6^ab^	8.56 ± 0.1^b‐i^	60.20 ± 0.1^hij^	45.29 ± 0.3^klm^
	*Grewia tembensis*	91.38 ± 1.0^ab^	8.14 ± 2.1^c‐j^	59.65 ± 1.5^h‐k^	53.62 ± 0.6^efg^
	*Ipomoea donaldsonii*	88.83 ± 1.6^ab^	6.04 ± 0.9^f‐j^	58.06 ± 0.7^ijk^	56.87 ± 1.0^efg^
	*Lannea rivae*	85.601.7 ± ^b^	12.13 ± 1.0^a‐e^	63.56 ± 0.6^c‐f^	46.39 ± 0.9^ijkl^
	*Rhus ruspolii*	90.65 ±0.9^ab^	4.55 ± 1.1^ij^	57.00 ± 0.8^kl^	47.35 ± 0.5^hijkl^
	*Tephrosia vogelii*	89.61 ± 1.2^ab^	12.18 ± 0.7^a‐e^	64.07 ± 0.5^b‐e^	44.42 ± 0.6^lm^
	Mean	90.01 ± 0.4	8.97 ± 0.6^b^	61.64 ± 0.6	48.40 ± 1.0^b^
Wet	*Acacia bussei*	87.87 ± 0.4^ab^	10.52 ± 0.6^a‐g^	64.10 ± 0.5^b‐e^	59.28 ± 0.2 cd
	*Acacia mellifera*	91.93 ± 0.2^ab^	10.24 ± 1.0^a‐h^	60.82 ± 0.6^fhi^	59.88 ± 0.1^c^
	*Acacia asak*	94.61 ± 1.6^a^	6.87 ± 0.3^e‐j^	60.77 ± 0.2^fhi^	44.00 ± 0.7^lmn^
	*Asepalum eriantherum*	89.60 ± 0.5^ab^	5.19 ± 0.3^g‐j^	57.00 ± 0.2^kl^	53.23 ± 0.3^fgh^
	*Balanites rotundifolia*	91.55 ± 1.6^ab^	10.99 ± 2.0^a‐f^	62.83 ± 1.4^def^	51.25 ± 0.9^ab^
	*Barleria spinisepala*	88.76 ± 2.7^ab^	7.35 ± 0.8^d‐j^	51.96 ± 0.1^m^	64.79 ± 2.0^ab^
	*Commiphora erythraea*	87.95 ± 0.2^ab^	11.83 ± 1.5^a‐e^	63.52 ± 1.1^c‐f^	54.70 ± 0.1^def^
	*Dalbergia microphylla*	91.78 ± 0.9^ab^	13.93 ± 0.6^ab^	65.33 ± 0.4^bcd^	50.90 ± 0.5^fghij^
	*Euclea divinorum*	88.29 ± 0.6^ab^	13.52 ± 0.8^abc^	66.64 ± 0.4^ab^	41.37 ± 0.3^mn^
	*Grewia evolute*	90.90 ± 1.2^ab^	8.47 ± 1.3^b‐i^	58.80 ± 0.8^ijk^	65.04 ± 0.9^a^
	*Grewia tembensis*	92.60 ± 1.7^ab^	15.00 ± 1.0^a^	63.96 ± 1.7^c‐e^	59.11 ± 1.1 cd
	*Ipomoea donaldsonii*	88.14 ± 1.5^ab^	4.91 ± 0.6^hij^	54.35 ± 0.4^lm^	52.40 ± 0.9^efg^
	*Lannea rivae*	86.67 ± 0.9^ab^	12.74 ± 0.6^a‐d^	60.36 ± 2.9^hij^	49.65 ± 0.5^ghijk^
	*Rhus ruspolii*	90.38 ± 2.5^ab^	10.61 ± 0.5^a‐g^	64.68 ± 1.1^b‐e^	51.11 ± 1.4^fghi^
	*Tephrosia vogelii*	89.62 ± 2.6^ab^	7.38 ± 0.6^d‐j^	61.88 ± 0.5^efh^	49.96 ± 1.5^ghijk^
	Mean	90.04 ± 0.5	9.97 ± 0.5^a^	61.13 ± 0.6	53.78 ± 1.0^a^
*p‐*Value	Feed species	0.0012	<0.0001	<0.0001	<0.0001
	Season	0.9549	0.0079	0.1453	<0.0001
	Interaction	0.3760	<0.0001	<0.0001	<0.0001

Season	Species	ADF (%DM)	NDF (%DM)	ADL (%DM)
Dry	*Acacia bussei*	38.97 ± 0.3^a^	61.88 ± 0.3^a^	14.37 ± 0.1^a^
	*Acacia mellifera*	22.30 ± 0.4^n^	38.21 ± 0.4^k^	5.75 ± 0.1^o^
	*Acacia asak*	24.67 ± 0.3^kimn^	41.90 ± 0.3^ijk^	6.76 ± 0.1^klm^
	*Asepalum eriantherum*	31.21 ± 0.3 cd	57.31 ± 0.3^bc^	10.17 ± 0.1^b^
	*Balanites rotundifolia*	30.23 ± 0.9^def^	56.01 ± 0.9^bcd^	9.33 ± 0.3^c^
	*Barleria spinisepala*	22.26 ± 0.3^n^	39.32 ± 0.3^k^	6.21 ± 0.1^mno^
	*Commiphora erythraea*	29.65 ± 0.5^defg^	54.57 ± 0.5^bcde^	8.76 ± 0.2^cde^
	*Dalbergia microphylla*	28.72 ± 0.3^efgh^	52.87 ± 0.3^bef^	8.35 ± 0.1^efgh^
	*Euclea divinorum*	35.38 ± 0.6^b^	58.77 ± 0.6^ab^	13.82 ±0.2^a^
	*Grewia evolute*	29.78 ± 0.2^defg^	54.92 ± 0.2^bcde^	8.54 ± 0.1^def^
	*Grewia tembensis*	25.27 ± 0.3^jklm^	43.75 ± 0.3^hij^	7.18 ± 0.1^jkl^
	*Ipomoea donaldsonii*	24.18 ± 0.4^lmn^	41.17 ± 0.4^ijk^	6.55 ± 0.1^lmn^
	*Lannea rivae*	29.05 ± 0.6^defgh^	54.18 ± 0.6^cde^	8.42 ± 0.2^defg^
	*Rhus ruspolii*	29.17 ± 0.3^defge^	50.91 ± 0.3^efg^	9.27 ± 0.1^c^
	*Tephrosia vogelii*	30.70 ± 0.4^cde^	54.68 ± 0.4^bcde^	9.41 ±0.1^c^
	Mean	28.77 ± 0.7^a^	50.70 ± 0.7^a^	8.86 ±0.4^a^
Wet	*Acacia bussei*	23.77 ± 0.1^lmn^	39.48 ± 0.2^jk^	6.22 ± 0.0^mno^
	*Acacia mellifera*	23.23 ± 0.0^mn^	38.56 ± 0.1^k^	5.98 ± 0.0^no^
	*Acacia asak*	30.36 ± 0.5^def^	55.61 ± 0.9^bcd^	9.11 ± 0.2 cd
	*Asepalum eriantherum*	25.70 ± 0.1^ijkl^	44.31 ± 0.3^hi^	7.33 ± 0.0^ijk^
	*Balanites rotundifolia*	27.64 ± 0.5^jhij^	47.23 ± 0.8^gh^	7.58 ± 0.1^ij^
	*Barleria spinisepala*	18.79 ±0.6^o^	33.21 ± 1.0^l^	4.58 ± 0.1^p^
	*Commiphora erythraea*	23.97 ± 0.1^lmn^	41.29 ±0.1^ijk^	6.67 ± 0.0^klmn^
	*Dalbergia microphylla*	27.77 ± 0.3^ghi^	48.80 ±0.5^fg^	7.66 ± 0.1^hij^
	*Euclea divinorum*	32.89 ±0.2^c^	57.82 ± 0.4^abc^	10.63 ± 0.1^b^
	*Grewia evolute*	19.17 ±0.3^o^	32.68 ±0.4^l^	4.72 ± 0.1^p^
	*Grewia tembensis*	23.76 ± 0.4^lmn^	40.20 ± 0.8^ijk^	6.41 ± 0.1^mno^
	*Ipomoea donaldsonii*	26.18 ± 0.5^ijkl^	46.82 ± 0.8^hg^	7.23 ± 0.1^jkl^
	*Lannea rivae*	27.96 ± 0.3^fghi^	49.11 ± 0.5^gh^	7.97 ± 0.1^fghi^
	*Rhus ruspolii*	26.81 ± 0.8^hijk^	47.23 ± 1.3^fg^	7.67 ± 0.2^hij^
	*Tephrosia vogelii*	28.12 ± 0.8^fghi^	48.68 ± 1.4^fg^	7.76± 0.2^ghij^
	Mean	25.74 ± 0.6^b^	44.74 ± 1.1^b^	7.17 ± 0.2^b^
*p‐*Value	Feed species	<0.0001	<0.0001	<0.0001
	Season	<0.0001	<0.0001	<0.0001
	Interaction	<0.0001	<0.0001	<0.0001

^abcd^Means with different superscripts in the same column are significantly different (*p* < .05)

Abbreviations: CP, crude protein; ADF, acid detergent fibre; NDF, neutral detergent fibre; OM, organic matter; RFV, relative feed value.

#### 3.2.2 Total ash and organic matter content

The total ash content of the analysed forage showed a significant variation (*p* < .01) across plant browse species and season. The ash content evaluated for camel feed resources significantly varied (*p* < .01) from 1.2% in *B*. *spinisepala* to 13.6% in *E. divinorum,* indicating lower OM content in favour of higher constituent of ash. The highest value of OM content were significant in *A*. *mellifera* (98.66%) and *C. erythraea* (95.31%) in the dry season followed by *Rhus ruspolii* (95.17%) and *E*. *divinorum* (95.02%) in the wet season. Excepting *Acacia bussei*, *A*. *asak*, *E*. *divinorum*, *R. ruspolii* and *Tephrosia vogelii*, all considered browse species showed significantly higher (*p* < .01) OM content during the dry season. In contrast, the OM content of *A*. *bussei* and *R*. *ruspolii* was significantly higher (*p* < .01) during wet season and lower in dry season. OM content of *A*. *asak*, *E*. *divinorum* and *T. vogelii* showed non‐significant (*p > *.05) variation at both seasons, indicating that meteorological variation does not affect the OM content of these species. Generally, the mean OM contents were significantly lower (*p* < .01) in the wet season (90.55%) though it showed some improvement during the dry season (92.33%). Similarly, Al‐Arif et al., (2017) and Chalchissa et al., (2014) reported 92.46% and 88.5% OM content for samples of mixed forage and green feed plants, respectively. Furthermore, Melaku et al., (2010) reported 91% average for mean OM content analysed from selected browse trees and shrub plant species, which corresponds with the result of this study.

#### 3.2.3 Crude protein

In the dry season, the CP content of the selected browse plant species varied (*p* < .01) from 7% in *E*. *divinorum* to 21.96% in *A*. *mellifera*. On the other hand, the CP content was ranged from 8.98% in *E*. *divinorum* to 24.27% and 23.56% in *Grewia evolute* and *B*. *spinisepala*, respectively, for the wet season. Except for *A*. *mellifera*, *L. rivae*, *A*. *asak* and *Ipomoea donaldsonii*, the CP contents of all considered browse species were significantly higher (*p* < .01) in the wet season samples (16.79%) than dry season samples (13.21%). Melaku et al., (2010) also reported a similar finding on the seasonal variation of CP content in browse feed resources utilized by camel, revealing 13.4% and 16.1% of mean CP during the dry and wet seasons, respectively. It showed also a significant (*p < *.01) variation across species. In opposite, the CP content of *I*. *donaldsonii* and *A*. *asak* species were significantly higher (*p* < .01) in the dry season. *A*. *mellifera* and *L*. *rivae* showed insignificant variation (*p* > .05).

All evaluated browse species CP contents at both seasons were higher than the required minimum level (7%) for ruminant feed intake and optimum rumen microbial functions (P. J. Van Soest, 1994). The minimum CP content of ruminant feed resources usually required for lactation and growth is 15% on a DM basis (Norton, 1982). Indeed, *B*. *spinisepala*, *G. tembensis*, *I. donaldsonii* and *A*. *mellifera*, in both seasons; *G*. *evolute*, *Asepalum eriantherum*, *A*. *bussei* and *C*. *erythraea* in the wet season and *A*. *asak* in the dry season had greater than 15% CP value on DM basis. The evaluated CP content of browse feed resource in both dry (7%–22%) and wet (9%–24%) seasons falls within the typical range of CP content (5%–50% CP on DM basis) in animal feedstuff, according to Galyean (2009). The mean annual CP content of analysed browse forage species was 15.12% on DM basis, which is less than 18.3% average CP content on DM basis in trees and shrub as reported by Dyness et al., (2013).

#### 3.2.4 Fibres

The NDF content of the analysed species ranged from 38.21% in *A*. *mellifera* to 61.88% in *A*. *bussei* during the dry season. Nonetheless, the amount varied from 32.68% in *G*. *evolute to* 57.82% in *E*. *divinorum* during the wet season (Table 3). The NDF content of evaluated browse species showed significant variation across species and seasons (*p < *.01) except for *A*. *mellifera*, *E*. *divinorum*, *G. tembensis* and *R*. *ruspolii*, which did not vary significantly (*p* < .01) following the season. Indeed, the result of this study indicated generally that significant higher NDF content of browse species was recorded in dry season with a mean of 50.65% against 44.65% in the wet season. *A*. *asak* and *I. donaldsonii* contained significantly higher (*p* < .01) NDF in the wet season.

The NDF content observed in this study is slightly higher than the findings of Melaku et al., (2010) who have revealed values ranging from 28.2% to 53.5% in dry and 28.9% to 58.6% in the wet seasons. The variations may arise due to differences in soil types and microclimatic conditions. The mean NDF value obtained during the wet season is in line with the report of Dyness et al., (2013) who showed a value of 44.85% for browse tree and shrub plants. Similarly, the mean NDF value of the examined feed resource at both seasons follows the reported range of 10%–80% NDF content of livestock feed resources (Galyean, 2009). Singh and Oosting (1992) classified feedstuffs with <45% NDF value DM basis as high‐ and medium‐quality forage, while those ranging from 45% to 65% are qualified as medium quality. Consequently, *B*. *spinisepala*, *G*. *tembensis* and *A*. *mellifera* can be classified as high‐quality forage species in both seasons. *G*. *evolute*, *A*. *eriantherum*, *A*. *bussei* and *C*. *erythraea* are considered as high‐quality roughage for the wet season, but *I*. *donaldsonii* and *A*. *asak* for the dry season. *R*. *ruspolii*, *B. rotundifolia*, *D. microphylla*, *T. vogelii*, *E. divinorum* and *L*. *rivae* are qualified as medium‐quality forage, as their NDF content has fallen within the range of 45%–65% at both seasons. The differences in NDF content between the browse species across season may arise due to cell wall accumulation in dry periods as a response to climatic variables.

ADF contents of the evaluated browse species varied from 22.26% in *A*. *mellifera* to 38.97% in *A*. *bussei* during the dry season and 18.79% in *B*. *spinisepala* to 32.89% in *E*. *divinorum* during the wet season. The current results indicated significant (*p* < .01) higher ADF amounts from the samples of dry season, showing low‐quality forage in this season, with generally lower amount in wet season, while only *A*. *asak* contained lower ADF during the dry season. Moreover, the results of this study showed observed significant variation between species, finding a lower ADF content of *B*. *spinisepala* and *A*. *mellifera* at both seasons. The mean ADF content is slightly higher than the average ADF content of 24.3% in dry season and 27.6% in the wet season (Melaku et al., 2010). In contrast, the ADF content in the current finding is lower than the value reported by Abebe et al., (2012) (40.6%–65.5% in dry season and 50.4%–57.3% during the wet season). *E*. *divinorum* and *A*. *bussei* contained higher ADF values in the dry season and qualified as poor‐quality and lower digestibility forage in such season, because feedstuffs with greater than 35% ADF content are considered as low‐quality roughage (Van Saun, 2006). Also, McDonald et al., (2002) have mentioned that ADF content and digestibility are negatively correlated. In effect, browse species examined in this study are less digestible at dry season in comparison to *A*. *asak*. Excepting *G*. *evolute* and *B*. *spinisepala* in wet season, all analysed roughage during both seasons contained ADF value surpassed the range level of 17%–21%, usually recommended for rumen stability (Garnsworthy et al., 2013; NRC, 2001).

This study revealed higher constituent levels of cellulose and hemicellulose and relatively lower contents. For all analysed browse species, except *I*. *donaldsonii and A*. *asak*, the hemicellulose content was higher in the dry season, indicating that forage quality is more likely affected by the season. However, the cellulose content was consistent in both seasons, excluding *A*. *asak*, which was higher in dry season.

During the dry season, lignin content varied from 5.75% in *A*. *mellifera* to 14.37% in *A*. *bussei*, whereas it ranged from 4.58% in *B*. *spinisepala* to 10.61% in *E*. *divinorum* during the wet season. Similarly, 5%–10% lignin on DM basis is the amount most often available in the roughage DM (Maynard et al., 1979). The mean lignin contents received lower value in the wet season (*p* < .01), apart from *A*. *mellifera*, *D. microphylla*, *I. donaldsonii* and *L. rivae,* which showed insignificant variation, indicating seasonal difference has not potential influence on these browse spaces. In reverse, *A*. *asak* contained significantly higher lignin in wet season (*p* < .01). Thus, almost all species indicated relatively a good forage quality in the wet season, except for *E*. *divinorum*. Excluding some values, the majority of evaluated browse species in this study contained much more than 5% lignin on DM basis in the dry season, which is the maximum level recommended for rumen stability (Garnsworthy et al., 2013). In addition, feedstuff with > 10% lignin content on DM basis negatively affects feed intake and digestibility (Barry et al., 1986; Waghorn et al., 1994).

#### 3.2.5 In vitro dry matter digestibility

The IVDMD of analysed browse species in this study ranged from 39.52% in *A*. *bussei* to 60.53% in *A*. *mellifera* during the dry season and varied from 41.37% in *E*. *divinorum* Hiern to 65.04% in *B*. *spinisepala* during the wet season. Generally, the mean IVDMD showed significant higher value for the foliage sampled in the wet season (*p* < .01), apart from *A*. *asak* which was more digestible in dry season, while the IVDMD of *A*. *mellifera*, *D. microphylla*, *E. divinorum*, *I. donaldsonii*, *L. rivae* and *R*. *ruspolii* was insignificantly varied with season (*p* < .01) (Table 3). Seasonal effect in IVDMD is in concordance with findings of Silva et al., (2017) and Abebe et al., (2012) in semi‐arid regions. The mean IVDMD content found for both seasons was lower than results of Melaku et al., (2010) in semi‐arid region of northern Ethiopia. This variation might be due to the relatively higher contents of cell wall components in analysed browse species of this study. On the other hand, IVDMD of ruminant feedstuffs varied due to dietary concentrations of feed resources, methods applied in laboratory analysis, season or period of the year, forage species, edible plant parts and stage of maturity (Abebe et al., 2012; Dambe et al., 2015; Hayirli et al., 2002; Mabjeesh et al., 2000; Melaku et al., 2010; Quansah & Makkar, 2012; Silva et al., 2017; Weaver et al., 1978).

According to Mosi and Butterworth (1985) criteria, *A*. *mellifera*, *B*. *spinisepala*, *G. tembensis* and *I*. *donaldsonii*, which contained >50% IVDMD on DM basis in both seasons, are qualified as a good‐quality forage species the entire year. However, the IVDMD content of *T. vogelii*, *E*. *divinorum* and *L*. *rivae* were <50% in both seasons, which categorizes them as a low‐quality roughage. The mean IVDMD observed in this study was comparable with the report of Mlay et al., (2006). The IVDMD observed in this study was lower than value reported by Tufarelli et al., (2010) (56%). This variation might be due to the relatively higher contents of cell wall components in analysed browse species of this study. According to Warne et al., (2010) and Rust and Rust (2013), some plant species were adapted and produced quality forage under hot and dry conditions; some are adapted to cooler and moist conditions and powerless to maintain their nutritional quality. The forage plants adapted and survived under high ambient temperature conditions, and water scarcity was low in quality (Bellard et al., 2012). Moreover, Sejian et al., (2016) reported that forage species adapted to hot and dried environmental conditions were more likely to have lower CP concentrations and high cell wall (lignin, cellulose and hemicellulose) contents, which qualify them as low quality and IVDMD.

#### 3.2.6 Ether extract

The EE value observed in this study varied from 2.71% in *B*. *rotundifolia* to 13.55% in *A*. *mellifera* during the dry season and varied from 4.91% in *I*. *donaldsonii* to 15% in *G*. *tembensis* during the wet season. The crude fat contents varied significantly (*p < *.01) thought forage species. For most considered browse species, the EE content varied insignificantly (*p* < .01) with season. However, the EE content of *B*. *rotundifolia*, *G*. *tembensis* and *R*. *ruspolii* was found to be the highest in wet season. Mean EE content observed in this study at both seasons was higher than the mean, 1.5% and 3.3% of tropical grasses and legume browse trees, respectively, and most similar to concentrates (9.7%) as cited by Mlay et al., (2006). The crude fat contents of all the investigated browse species in both seasons fell within the range of 1%–20% EE on DM basis, the amount often found in livestock feedstuff (Galyean, 2009).

### 3.3 Forage quality evaluation of browse species

#### 3.3.1 Total digestible nutrient

The TDN value ranged from 57% in *R*. *ruspolii* to 69.23% in *A*. *mellifera* during the dry season and from 51.96% in *B*. *spinisepala* to 66.64% in *E*. *divinorum* during the wet season with a mean TDN value of 61.4%. The TDN of *A*. *mellifera*, *B. spinisepala*, *I. donaldsonii and L. rivae* was significantly higher (*p* < .01) in the dry season. On the other hand, the TDN of *B*. *rotundifolia*, *E. divinorum*, *G. tembensis* and *R. ruspolii* was found to be the highest at the wet season (Table 3).

According to Rivera and Parish (2010), feedstuff that contained below 52% TDN on DM basis limited feed intake and resulted in poor livestock performance. Subsequently, all the investigated browse species can be qualified as adequate foods for livestock as it contained > 52% TDN on DM basis in both seasons. The mean TDN obtained in both season was higher than the reported value (46.5%) by Mlay et al., (2006) on tropical browse species. The variation in TDN might be due to difference in the evaluated forage species, in vitro digestion method, the equation used to calculate and the environmental factors. According to Ball et al., (2001), the quality of animal feedstuff is often affected by the differences in forage species, environmental temperature and maturity stage.

#### 3.3.2 Relative feed value of the browse species

RFV observed in this study ranged from 88.02 for *A*. *bussei* to 174.25 for *A*. *mellifera* in dry season, whereas it varied from 101.82 for *E*. *divinorum* to 210.65 for *G*. *evolute* in wet season (Table 3). The RFV of almost considered browse species were significant higher (*p* < .01) in wet season excluding *A*. *mellifera*, *D. microphylla*, *E. divinorum* and *R. ruspolii*, which did not changed significantly (*p* < .01) among seasons. Contradictory, the RFV of *A*. *asak* and *I*. *donaldsonii* was highest at the dry season.

*A. mellifera* and *B. spinisepala* were the browse species with best forage quality in both seasons because animal feedstuffs with above 151 RFV are recognized as a prime quality roughage (Rivera & Parish, 2010). The forage qualities and nutritional values of forage plants vary among fodder plant species (Ball et al., 2001). According to Rivera and Parish (2010), feedstuffs with greater than 17% CP, below 35% ADF, below 45% NDF, above 125 RFV and 60% TDN were considered as good‐quality forage. Plant parts accessible to different browse livestock species showed no significant variation in chemical composition. Moreover, Sanon (2007) reported significant variation of chemical composition and quality of the browse species such as *Guiera senegalensis*, *Pterocarpus lucens and Acacia senegal*. However, it seems that *E*. *divinorum* in both seasons and *A*. *bussei* in the dry season are not preferred as roughage because their RFV fell to third level between 87 and 102 according to Rivera and Parish (2010).

The CP and IVDMD had shown a significant positive correlation with RFV (Table 4). Oppositely, the RFV and IVDMD of the browse species were negatively correlated with NDF, ADF and lignin contents of analysed feedstuff. Forage quality improved with increasing CP contents of feedstuffs (Rivera & Parish, 2010). Similarly, NRC (2001) revealed declining forage quality with higher levels of NDF contents on DM basis.

**TABLE 4:** Correlation of relative feed value and in vitro dry matter digestibility with chemical compositions of browse species

Feed chemical composition	RFV		IVDMD	
	*R*	*p*‐value	*R*	*p*‐value
CP	0.98***	<.01	1.00***	<.01
ADF	−0.95***	<.01	−0.95***	<.01
NDF	−0.99**	<.01	−1.00***	<.01
TDN	0.65**	<.01	0.70***	<.01
ADL	−0.88***	<.01	−0.89***	<.01
EE	−0.12*^NS^*	.68	−0.092*^NS^*	.74
IVDMD	0.98***	<.01		

Abbreviation: NS, non‐significant

** *p* < .05

*** *p* < .01

## 4 CONCLUSION

This study showed that chemical composition and feed quality of examined browse species were found to be significantly varied (*p* < .01) across seasons. Browse species that meet the prime quality standard were *G*. *evolute*, *G*. *tembensis*, *A*. *bussei*, *C*. *erythraea*, *I. donaldsonii*, *A*. *mellifera*, *B. spinisepala* and *A*. *asak*. These species can be also used as multi‐purpose plants in agroforestry system of the semi‐arid regions, like fodder banks and live fences, as well as for soil conservation. *A*. *mellifera* and *B. spinisepala* are good potential forage in both seasons, while *I*. *donaldsonii* and *A*. *asak* are better to be harvested in the dry season. On the other hand, *G*. *evolute*, *G*. *tembensis*, *A. bussei* and *C*. *erythraea* are better used in the wet season. At richest level on vital components (*CP*, *NDF*, *ADF* and *ADL*), these species can serve as well ruminant diets, like for camel. Further investigations based on animal trials are needed in order to confirm the classification standards of feed quality used in this study.

## CONFLICT OF INTEREST

No potential conflicts of interest to declare.

## AUTHOR CONTRIBUTION

**Matiwos Habte:** Data curation; Funding acquisition; Investigation; Methodology; Software; Writing‐original draft; Writing‐review & editing. **Mitiku Eshetu:** Supervision; Validation; Visualization; Writing‐review & editing. **Dereje Andualem:** Data curation; Formal analysis; Methodology; Software; Supervision; Validation; Visualization; Writing‐review & editing. **Melesse Maryo:** Methodology; Resources; Visualization; Writing‐review & editing. **Abiyot Legesse:** Data curation; Formal analysis; Project administration; Software; Supervision; Validation; Writing‐review & editing.

## ANIMAL WELFARE STATEMENT

The authors confirm that the ethical policies of the journal, as noted on the journal's author guidelines page, have been adhered to. No ethical approval was required as this is an original data with no animals used for scientific purpose.

### PEER REVIEW

The peer review history for this article is available at https://publons.com/publon/10.1002/vms3.471.

## Data Availability

The authors declare that all data supporting the findings of this study are available within the article and its supplementary information files.
